# Supplementary material for: Structural basis for DNA 5´-end resection by RecJ
Source: eLife. 2016 Apr 8;5:e14294. doi: 10.7554/eLife.14294 (PMC4846377; doi:10.7554/eLife.14294)
Supplement: Supplementary file 1. — DOI: http://dx.doi.org/10.7554/eLife.14294.023 [file elife-14294-supp1.docx]

**Supplementary file 1: Primers used for cloning and mutagenesis**

| **Primers** | **Sequence (5′→3′)** |
| --- | --- |
| RecJ_F (NdeI) | TTTTTTCATATGAGCCGGCCTGCCCACTGG |
| RecJ_core_F (NdeI) | TTTTTTCATATGCTAGAACTGACGCCCAACCCGG |
| RecJ_R (BamHI) | TTTGGATCCTTAACCCGCAGCGAGCGCCAG |
| RecJ_core_R (BamHI) | TTTGGATCCTCAAGCCGGGACCGGGGTCGG |
| RecJ_ΔC_R (BamHI) | TTTGGATCCTCAGCGCAGCGCCGCCGCGT |
| RecJ_ΔαCT_R (BamHI) | TTTGGATCCTCACGGTACAGGCAGCCCGAGCATC |
| RecJ_Y80A_F | CCCCGTCGGCGTCGGCGTCACCGTGAATAC |
| RecJ_Y80A_R | GTATTCACGGTGACGCCGACGCCGACGGGG |
| RecJ_R109A_F | GTAGCCCTCGTTCAATGCGTGGGGAATGAACCCG |
| RecJ_R109A_R | CGGGTTCATTCCCCACGCATTGAACGAGGGCTAC |
| RecJ_Y114A_F | GTCAGGATGAATCCCGGCGCCCTCGTTCAATCGG |
| RecJ_Y114A_R | CCGATTGAACGAGGGCGCCGGGATTCATCCTGAC |
| RecJ_V224A_F | GCAGCGGCGCCGCGTCGGCCACC |
| RecJ_V224A_R | GGTGGCCGACGCGGCGCCGCTGC |
| RecJ_F269A_F | CGGCGCGAGGATAGCGGCCACGTCACGC |
| RecJ_F269A_R | GCGTGACGTGGCCGCTATCCTCGCGCCG |
| RecJ_R280A_F | CGCCTCCCCCATCGCCCCAGCCGCATTG |
| RecJ_R280A_R | CAATGCGGCTGGGGCGATGGGGGAGGCG |
| RecJ_R313A/R314A_F | AACATATCGTCCTGAATCTTGGCCGCCTCCTGGTTGCGAATTTCCAG |
| RecJ_R313A/R314A_R | CTGGAAATTCGCAACCAGGAGGCGGCCAAGATTCAGGACGATATGTT |
| RecJ_K353A_F | ACGTCTCCACCAGCGCGCTCGCCACGATGC |
| RecJ_K353A_R | GCATCGTGGCGAGCGCGCTGGTGGAGACGT |
| RecJ_S371A_F | TGGAGCGCACCGCGCCTTTGCCCTG |
| RecJ_S371A_R | CAGGGCAAAGGCGCGGTGCGCTCCA |
| RecJ_R373A_F | GCCGGGGGTGGAGGCCACCGAGCCTTTG |
| RecJ_R373A_R | CAAAGGCTCGGTGGCCTCCACCCCCGGC |
| RecJ_R393A_F | TGCCCGCCGAAGGCCCCCAGGAGGTC |
| RecJ_R393A_R | GACCTCCTGGGGGCCTTCGGCGGGCA |
| RecJ_H397A_F | CCGCGCCGGGGGCCCCGCCGAAGC |
| RecJ_H397A_R | GCTTCGGCGGGGCCCCCGGCGCGG |
| RecJ_R475A_F | GCTTGCCCACCAATGCGGTGTCAGTCAGCG |
| RecJ_R475A_R | CGCTGACTGACACCGCATTGGTGGGCAAGC |
| RecJ_V477A_F | GCCCTGCTTGCCCGCCAATCGGGTGTC |
| RecJ_V477A_R | GACACCCGATTGGCGGGCAAGCAGGGC |
| RecJ_Y496_F | TCGCGTTCGCTGGCTTTCATGCCTTTGACGCCACCG |
| RecJ_Y496_R | CGGTGGCGTCAAAGGCATGAAAGCCAGCGAACGCGA |
| RecJ_W517A_F | GGTCCGGCCCTTCGCCTCGTTGAGGGCA |
| RecJ_W517A_R | TGCCCTCAACGAGGCGAAGGGCCGGACC |
| RecJ_D79A_F | CGTCGGCGTCGTAGGCACCGTGAATACGG |
| RecJ_D79A_R | CCGTATTCACGGTGCCTACGACGCCGACG |
| RecJ_D81A_F | CCCCGTCGGCGGCGTAGTCACCG |
| RecJ_D81A_R | CGGTGACTACGCCGCCGACGGGG |
| RecJ_D83A_F | ACTACGACGCCGCCGGGGTGAGTGC |
| RecJ_D83A_R | GCACTCACCCCGGCGGCGTCGTAGT |
| RecJ_D135A_F | GGACACCCCACAGGCCACCGTGACCAC |
| RecJ_D135A_R | GTGGTCACGGTGGCCTGTGGGGTGTCC |
| RecJ_D223A_F | GCGGCGCCACGGCGGCCACCGTG |
| RecJ_D223A_R | CACGGTGGCCGCCGTGGCGCCGC |
| RecJ_D158A_F | GCGCGTGGTGGGCGGTGACCACG |
| RecJ_D158A_R | CGTGGTCACCGCCCACCACGCGC |
| RecJ_H159A_F | CCCGGCGCGTGGGCGTCGGTGACCAC |
| RecJ_H159A_R | GTGGTCACCGACGCCCACGCGCCGGG |
| RecJ_H160A_F | TCGCCCGGCGCGGCGTGGTCGGTGAC |
| RecJ_H160A_R | GTCACCGACCACGCCGCGCCGGGCGA |
