# Supplementary material for: Structural basis for DNA 5´-end resection by RecJ
Source: eLife. 2016 Apr 8;5:e14294. doi: 10.7554/eLife.14294 (PMC4846377; doi:10.7554/eLife.14294)
Supplement: Supplementary file 2. — DOI: http://dx.doi.org/10.7554/eLife.14294.024 [file elife-14294-supp2.docx]

**Supplementary file 2: Strains and plasmids used in this study**

| **Strain and plasmid** | **Description** | **Source** |
| --- | --- | --- |
| **Strains** |  |  |
| ***E.coli*** |  |  |
| DH5α | *E. coli* cloning strain | Laboratory stock |
| Rosetta (DE3) | *E. coli* expression strain | Laboratory stock |
| ***D.radiodurans*** |  |  |
| R1 | Wild-type strain, ATCC 13939 | Laboratory stock |
| *ΔrecJ* | As R1 but *recJ::str* | Laboratory stock |
| *ΔrecJ/pk-recJ* | As *ΔrecJ* but compensated with pk-*recJ* | Laboratory stock |
| *ΔrecJ/pk-recJΔCα* | As *ΔrecJ* but compensated with pk-*recJΔCα* | This study |
| **Plasmids** |  |  |
| pMD18-T vector | For TA cloning | Laboratory stock |
| pET28-HMT | Modified from pET28a containing 6XHis-tag, maltose binding protein and TEV protease site | Laboratory stock |
| HMTJ | As pET28-HMT but ligated with *drrecJ* | Laboratory stock |
| HMTJcore | As pET28-HMT but ligated with *drrecJcore* | This study |
| HMTJΔC | As pET28-HMT but ligated with *drrecJΔC* | This study |
| HMTJΔCα | As pET28-HMT but ligated with *drrecJΔCα* | This study |
| HMTSSB | As pET28-HMT but ligated with *drssb* | Laboratory stock |
| HMTSSBΔC | As pET28-HMT but ligated with *drssbΔC* | This study |
| HMTRecQ | As pET28-HMT but ligated with *drrecQ* | This study |
| pRADK | *E. coli-D. radiodurans* shuttle vector | Laboratory stock |
| pk-*recJ* | As pRADK but *kan^r^* was replaced with *drrecJ* gene | Laboratory stock |
| pk-*recJΔCα* | As pRADK but *kan^r^* was replaced with *drrecJΔC****α*** fragment | This study |
