# Supplementary material for: Structural basis for DNA 5´-end resection by RecJ
Source: eLife. 2016 Apr 8;5:e14294. doi: 10.7554/eLife.14294 (PMC4846377; doi:10.7554/eLife.14294)
Supplement: Supplementary file 3. — DOI: http://dx.doi.org/10.7554/eLife.14294.025 [file elife-14294-supp3.docx]

**Supplementary file 3: DNA substrates used in this study**

|  | **Sequence (5′→3′)** | **Label** |
| --- | --- | --- |
| **Crystallization** |  |  |
| KY01 (complex II) | GATGTACGCTAGGCACGC | None |
| KY02 (complex III) | CTGATGGCA | None |
| **Nuclease assays** |  |  |
| KY03 (ssDNA) | TTTTTTTTTTTTTTTTTTTTTTTTTTTTTTAGAGCACTC  GAGTACT | 3′ FAM |
| KY04 (5′ overhang) | TTTTTTTTTTTTTTAGTACTCGAGTGCTGGCGAGCAC  TCGAGTACT | 3′ FAM |
| KY05 (blunt end) | AGTACTCGAGTGCTGGCGAGCACTCGAGTACT | 3′ FAM |
| KY06 (3′ overhang) | CGTCTAGACAGCTCACCCGGCCGGGTGAGCTGTCTA  GACGGTCAGC | 3′ FAM |
| KY07 (ssRNA) | AUAAGAGGAGAAGGCGAG | 3′ FAM |
| KY08 (poly(dA)) | AAAAAAAAAAAAAAAAAAAA | 3′ FAM |
| KY09 (poly(dT)) | TTTTTTTTTTTTTTTTTTTT | 3′ FAM |
